# LIFE COURSE PATHWAYS OF CHILDHOOD SOCIOECONOMIC STATUS TOWARD CLINICAL RISK FACTORS FOR WORSE KIDNEY FUNCTION

**DOI:** 10.1093/geroni/igz038.1272

**Published:** 2019-11-08

**Authors:** Agus Surachman, Alexis Santos, Jonathan Rush, Lacy Alexander, Christopher Coe, David M Almeida

**Affiliations:** 1 The Pennsylvania State University, University Park, Pennsylvania, United States; 2 University of Victoria, Victoria, British Columbia, Canada; 3 University of Wisconsin - Madison, Madison, Wisconsin, United States

## Abstract

This study examines the roles of daily stress processes as a possible mediator of how life course socioeconomic inequality is reproduced in day-to-day experiences and creates disparities in clinical risk for rapid kidney function declines in adulthood. Data are from 1174 middle and older adults (56% female; ages 40–84, Mage = 56.2) who participated in the MIDUS study wave 2 and Refresher. We found significant pathways that childhood SES was associated with education and adult SES, adult SES was associated with exposure to daily stressors and daily stressor reactivity to negative affect, positive affect, and somatic symptom. In turn, higher report of daily somatic symptom and lower report of daily positive affect were associated with higher CKD risk factors. Finally, childhood SES was directly associated with CKD risk factors. Childhood SES is associated with clinical risks for rapid kidney function decline through direct association and life course accumulation.

